# Multi-omics identification and validation of oxidative phosphorylation–related hub genes in schizophrenia

**DOI:** 10.3389/fgene.2025.1690947

**Published:** 2025-10-23

**Authors:** Yu Zhou, Shuang Zhang, Yao-Xia Liu, Xin Dai, Ting Zhang, Xiao-Tao Xu, Sheng-Nan Deng, Min-Yan Yang, Zhen Fan

**Affiliations:** ^1^ Department of Psychological Health, The Second People’s Hospital of Yibin, Yibin, China; ^2^ Department of Geriatrics, Affiliated Hospital of Southwest Medical University, Luzhou, China; ^3^ Department of Geriatrics, Sichuan Provincial People’s Hospital, University of Electronic Science and Technology of China, Chengdu, China; ^4^ Department of Neurology, The Fourth People’s Hospital of Chengdu, Chengdu, China

**Keywords:** schizophrenia, oxidative phosphorylation, mitochondrial dysfunction, multi-omics, single-nucleus RNA sequencing

## Abstract

**Introduction:**

Dysfunction in mitochondrial oxidative phosphorylation (OXPHOS) has been implicated in the pathophysiology of schizophrenia, yet its molecular underpinnings remain poorly defined. In this study, we performed an integrative multi-omics analysis to delineate these molecular signatures.

**Methods:**

Bulk transcriptomic datasets of schizophrenia patients and controls were obtained from the Gene Expression Omnibus. Differentially expressed genes (DEGs) associated with OXPHOS were identified through a combination of differential expression analysis, single-sample gene set enrichment analysis (ssGSEA), and weighted gene co-expression network analysis (WGCNA). Hub genes were prioritized by machine learning algorithms (LASSO, SVM-RFE, and random forest). These hub genes were validated using an independent dataset and further corroborated by RT-qPCR in an MK-801-induced mouse model. Single-nucleus RNA sequencing (snRNA-seq) was employed to delineate cell type-specific oxidative phosphorylation activity and transcriptional profiles.

**Results:**

Transcriptomic analysis identified 130 DEGs between schizophrenia and controls, significantly enriched in oxidative phosphorylation and mitochondrial respiration pathways. Subsequent ssGSEA confirmed the reduced OXPHOS enrichment scores in schizophrenia. Furthermore, WGCNA uncovered two hub modules significantly associated with OXPHOS, which also showed strong correlations with schizophrenia. Intersecting their 2,609 module genes with 130 DEGs yielded 69 OXPHOS-related DEGs. From these, machine learning prioritized six hub genes, four of which demonstrated strong diagnostic potential and robust correlations with OXPHOS scores. Extending these findings *in vivo*, MK-801–treated mice exhibited behavioral and neuronal deficits, reduced ATP5A fluorescence intensity, and decreased ATP concentrations; expression of all four hub genes was significantly altered, with three (MALAT1, PPIL3, and ITM2A) concordant with transcriptomic results. Finally, snRNA-seq analysis indicated that OXPHOS is the principal ATP-generating pathway in the brain, with notable enrichment in excitatory neurons and endothelial cells, and further revealed significant correlations of MALAT1, PPIL3, and ITM2A with OXPHOS, consistent with bulk and *in vivo* observations.

**Conclusion:**

This finding suggests a potential link between OXPHOS dysfunction and schizophrenia, with MALAT1, PPIL3, and ITM2A emerging as candidate regulators of this process.

## Introduction

Schizophrenia is a chronic psychiatric disorder characterized by hallucinations, cognitive impairments, and negative mood, affecting ∼1% of the global population and ranking among the leading causes of disability worldwide. Despite decades of research, major questions remain unanswered. Current treatments predominantly target dopaminergic pathways to alleviate positive symptoms, but they demonstrate limited efficacy in addressing cognitive dysfunction and negative symptoms (Wu Q. et al., 2021), highlighting a critical unmet clinical need. Recent evidence increasingly implicates mitochondrial dysfunction and disrupted energy metabolism in the pathophysiology of schizophrenia (Zilocchi et al., 2020; Ni et al., 2024).

Oxidative phosphorylation (OXPHOS), the principal mitochondrial mechanism responsible for adenosine triphosphate (ATP) synthesis, is essential for neuronal functions including action potential propagation, synaptic transmission, and synaptic plasticity. Neuroimaging studies consistently report reduced ATP levels in the prefrontal, temporal, and frontal cortices of schizophrenia patients, correlating with cognitive deficits and negative symptoms (Duarte and Xin, 2019; Pruett and Meador-Woodruff, 2020). Postmortem transcriptomic analyses further reveal downregulation of OXPHOS-related genes and respiratory chain enzymes in the dorsolateral prefrontal cortex, particularly within parvalbumin-positive interneurons, which are essential for cortical synchrony and cognitive control (Sullivan et al., 2019; Morén et al., 2025). Complementary *in vivo* spectroscopy and molecular profiling investigations demonstrate that OXPHOS deficits are concomitant with abnormal lactate accumulation, redox imbalance, and dysregulated inflammatory responses in the prefrontal cortex (Enwright Iii et al., 2018; Ľupták et al., 2021; Fizíková et al., 2023), characteristics that are widely acknowledged as pathological hallmarks of schizophrenia. These findings collectively suggest that OXPHOS dysfunction may represent a potential metabolic alteration in schizophrenia. However, the mechanisms linking mitochondrial deficits to the progression of the disease are not yet well understood.

Recent advances in high-throughput sequencing technologies have significantly expanded our understanding of disease mechanisms. In schizophrenia, previous transcriptomic and proteomic analyses have identified numerous differentially expressed genes and proteins (Steiner et al., 2017; Khan et al., 2023). However, investigations specifically focusing on OXPHOS remains limited and often confined to single datasets, leaving its mechanistic contribution poorly defined. In this study, we focused on OXPHOS by integrating bulk transcriptomics, single-nucleus RNA sequencing (snRNA-seq), and the MK-801 mouse model. This integrative approach suggests a potential mechanistic link between OXPHOS dysfunction and schizophrenia and highlights three hub genes that may mediate this association. These findings may advance the understanding of schizophrenia pathophysiology and provide a basis for future therapeutic development.

## Materials and methods

### Data acquired

The NCBI GEO database (https://www.ncbi.nlm.nih.gov/geo/) was queried using the terms “Schizophrenia” and “*Homo sapiens*” to obtain expression profiles from dorsolateral prefrontal cortex tissue. Three datasets were retrieved. The datasets GSE87610 (GPL13667) and GSE53987 (GPL570) encompass bulk microarray expression data. Specifically, GSE87610, which includes 65 samples from individuals with schizophrenia and 72 control samples, was utilized as the training set. In contrast, GSE53987, comprising 15 schizophrenia samples and 19 control samples, was employed as the validation set. Because of its limited sample size, the validation set was used only for expression validation. Additionally, as no snRNA-seq data from schizophrenia brain tissue were publicly available, GSE247416 (GPL24676), consisting of snRNA-seq data from the dorsolateral prefrontal cortex of 37 neurologically healthy adults, was analyzed to enable cell type–specific transcriptomic characterization.

### Differential gene expression analysis and functional annotation

Differential expression analysis was performed using the limma package (Ritchie et al., 2015). Expression data were log2-transformed and normalized, and linear models were fitted with lmFit followed by empirical Bayes moderation using eBayes. Genes with |log2FC| > 0.5 and unadjusted P < 0.05 were defined as differentially expressed genes (DEGs). Heatmaps were generated using the pheatmap package (Wickham and Sievert, 2009). Gene Ontology Biological Process (GO-BP) and Kyoto Encyclopedia of Genes and Genomes (KEGG) enrichment analyses were performed with a significance threshold of P < 0.05 (Wu T. et al., 2021).

### Single-sample gene set enrichment analysis (ssGSEA)

The OXPHOS gene set, comprising 132 marker genes, was obtained from the Molecular Signatures Database (MSigDB; https://www.gsea-msigdb.org/gsea/msigdb) and is listed in Supplementary Table S1. Pathway enrichment scores for each sample were calculated using ssGSEA (Hänzelmann et al., 2013). Statistical differences between groups were evaluated using the Wilcoxon rank-sum test, with significance established at a threshold of P < 0.05.

### Weighted correlation network analysis (WGCNA)

WGCNA was performed with the WGCNA package (Langfelder and Horvath, 2008). Outlier samples were excluded, and the optimal soft-thresholding power was determined from the scale-free topology fit index (signed R^2^) and mean connectivity, with a power of 2 selected. Co-expression modules were identified using a dynamic tree-cutting algorithm with a minimum module size of 100 and a merging threshold of 0.1. Pathway enrichment scores were used as phenotypic traits, and Pearson correlation analysis was performed with the psych package (Lin et al., 2023). Modules showing the strongest positive and negative correlations with OXPHOS scores were designated as OXPHOS-associated modules. In addition, Module–trait relationships were also examined to assess correlations with schizophrenia status. Genes from OXPHOS-associated modules were then intersected with DEGs to identify OXPHOS-related DEGs for downstream analyses.

### Machine learning and validation analyses

To refine disease-associated candidates, three machine learning algorithms: least absolute shrinkage and selection operator (LASSO) regression (Friedman et al., 2010), support vector machine–recursive feature elimination (SVM-RFE) (Cinelli et al., 2017), and random forest (RF) (Breiman, 2001), were applied to the training dataset GSE87610. Genes identified by all methods were considered signature hub genes. Receiver operating characteristic (ROC) curve analysis determined genes with AUC >0.7 and P < 0.05 as diagnostically significant (Robin et al., 2011). Hub gene expression was validated in both the GSE87610 and GSE247416 cohorts using the Wilcoxon rank-sum test (P < 0.05). Correlation with OXPHOS scores was also assessed, with P < 0.05 as significant.

### snRNA-seq data analysis

The snRNA-seq data were processed using the Seurat R package (Satija et al., 2015). Cells with 500–30,000 detected genes and <10% mitochondrial transcripts were retained. After quality control, 799,217 cells were included for downstream analysis. Data were normalized, and highly variable genes were identified. Dimensionality reduction was performed by principal component analysis (PCA), followed by sample integration. Cell clustering was conducted with FindNeighbors and FindClusters (Stuart et al., 2019) and cell types were annotated based on canonical marker genes from published studies (Fröhlich et al., 2024). Pathways associated with ATP synthesis, including OXPHOS, glycolysis/gluconeogenesis, and the tricarboxylic acid (TCA) cycle, were annotated utilizing KEGGREST (Tenenbaum and Maintainer, 2021). Enrichment scoring was conducted using AUCell (Aibar et al., 2017). Subsequently, OXPHOS scores were calculated employing multiple complementary methodologies, including AUCell, AddModuleScore, Scoring, singscore, ssGSEA, and UCell (Tirosh et al., 2016; Aibar et al., 2017; Foroutan et al., 2018; DeTomaso et al., 2019; Andreatta and Carmona, 2021; Jin et al., 2021). Spearman correlation analysis was conducted to evaluate the associations between OXPHOS enrichment scores and the expression of hub genes. CellChat was employed to infer signaling networks among various cell types (Azadian et al., 2023). Cell trajectory analysis was performed with the Monocle package (Joo et al., 2024) using the top 2,000 highly variable genes to reconstruct lineage trajectories across cell subpopulations. This approach enabled pseudotime inference for key cell types and dynamic profiling of hub gene expression along cellular state transitions.

### Animal procedures

N-methyl-D-aspartate receptor (NMDAR) hypofunction is a major hypothesis in schizophrenia pathophysiology, and MK-801, a non-competitive NMDAR antagonist, is widely used to establish corresponding animal models (Ang et al., 2021). Five-week-old male C57BL/6 mice were used. Following a 1-week acclimatization, animals were randomly assigned to either the control or MK-801 group (n = 6 per group) and housed under standard conditions with *ad libitum* access to food and water. MK-801 (HY-15084, MCE, China) was dissolved in 0.9% saline and administered intraperitoneally at 0.5 mg/kg (10 ml/kg) once daily for 14 days; controls received an equal volume of saline. Behavioral assessments, including the open-field and novel object recognition tests, were conducted 2 days after the final injection. Mice were subsequently anesthetized with isoflurane and euthanized by cervical dislocation. Bilateral prefrontal cortices were rapidly dissected: one hemisphere was fixed in 4% paraformaldehyde for histology, and the other was snap-frozen in liquid nitrogen and stored at −80 °C for molecular analyses. All animal procedures were approved by the Ethics Committee of Southwest Medical University (No. 20250403-005).

### Open field test

Locomotor activity was evaluated using the open-field test, a standard paradigm for assessing hyperactivity analogous to the positive symptoms in schizophrenia models. Mice were individually placed in a black open-field chamber (50 × 50 × 50 cm), and locomotion was recorded for 5 min using EthoVision XT software (Noldus, Netherlands). The total distance traveled served as the primary measure of locomotor activity. Chambers were cleaned with 75% ethanol and air-dried between trials.

### Novel object recognition test

The novel object recognition test consisted of training and testing phases, both conducted in an open-field chamber. During training, two identical objects (A1 and A2) were placed in adjacent corners, 5 cm from the walls. Mice were placed facing away from the objects and allowed to explore for 5 min. After a 6-h interval, A2 was replaced with a novel object (B), and exploration time for A1 and B was recorded during a subsequent 5-min session. Recognition memory was quantified using the discrimination index (DI = [TB − TA1]/[TB + TA1]), where TA1 and TB represent the time spent exploring the familiar and novel objects, respectively; lower DI values indicated impaired recognition memory. Chambers and objects were cleaned with 75% ethanol between sessions, and mice had no prior exposure to the objects or arena.

### Nissl staining

Nissl staining was performed to identify dark neurons in the prefrontal cortex. Brain tissues were fixed, embedded in paraffin, sectioned, and stained with cresyl violet for examination under a light microscope. Dark neurons were characterized by intense basophilic staining, shrunken soma, and condensed or fragmented nuclei, distinct from normal neurons. Manual counting under the microscope quantified these neurons. Three random, non-overlapping regions were analyzed in each section, and quantification was independently performed by two blinded investigators.

### Immunofluorescence

Tissue sections were permeabilized and blocked using 10% goat serum for 1 h at room temperature. This was followed by an overnight incubation at 4 °C with an anti-ATP5A primary antibody (14676-1-AP, Proteintech, China). Subsequently, sections were washed and incubated for 1 h with Alexa Fluor 488–conjugated goat anti-mouse IgG. Nuclei were counterstained with DAPI. The slides were then mounted and visualized using a fluorescence microscope (OLYMPUS VS200, Japan). Image analysis was conducted using OlyVIA and ImageJ software. For each section, three random fields within the prefrontal cortex were captured, and the mean fluorescence intensity of ATP5A was quantified after subtracting background fluorescence ( $I_{\text{corrected}}=I_{\text{raw}}-I_{\text{background}}$ ). The quantification process was independently carried out by two investigators who were blinded to the experimental conditions.

### ATP content detection

Brain tissues were homogenized, and ATP levels were quantified using an ATP Content Detection Kit (BC0300, Solarbio, Beijing) following the manufacturer’s protocol. Absorbance was measured at 340 nm with a microplate reader.

### RT-qPCR

Total RNA was extracted using TRIzol reagent, and cDNA was synthesized with the PrimeScript RT kit (RR037B, Takara, Japan). Quantitative PCR was performed using SYBR Green Premix (HY-K0524, MCE, China) with 2 µL of cDNA per reaction. Primers (Table 1) were designed and synthesized by Beijing Tsingke Biotech Co., Ltd. GAPDH was used as the internal control for the protein-coding genes PPIL3, ITM2A, and GJA1, whereas 18S rRNA served as the reference for the long non-coding RNA MALAT1. Relative expression levels were calculated using the 2^−^ΔΔ^Ct method. All reactions were performed in triplicate, and fold changes were expressed relative to the control group (set as 1).

**TABLE 1:** Sequence of primers for RT-qPCR.

Genes	Forward primer 5′-3′	Reverse primer3′-5′
18S rRNA	agg​gga​gag​cgg​gta​aga​ga	gga​cag​gac​tag​gcg​gaa​ca
MALAT1	ggg​agt​ggt​ctt​aac​agg​gag​gag	aac​agc​ata​gca​gta​cac​gcc​ttc
GAPDH	agg​tcg​gtg​tga​acg​gat​ttg	tgt​aga​cca​tgt​agt​tga​ggt​ca
PPIL3	gaa​cac​cca​aaa​cat​gtg​aga​a	tga​acc​atg​aag​ccc​ttg​ata​t
ITM2A	ttc​tga​gga​tcc​tgt​caa​ttc​c	tca​aag​tcg​tga​ata​att​gcc​g
GJA1	cac​tct​cac​cta​tgt​ctc​ctc​ctg	cgc​tgg​ctt​gct​tgt​tgt​aat​tg

### Statistical analysis

Bioinformatic analyses were conducted in R 4.2.2. For *in vivo* validation, intergroup differences were evaluated using unpaired two-tailed t-tests in SPSS 23, with statistical significance set at P < 0.05. Data visualization was performed using GraphPad Prism 9.

## Results

### Differential expression and enrichment analyses reveal OXPHOS downregulation in schizophrenia

**FIGURE 1:**
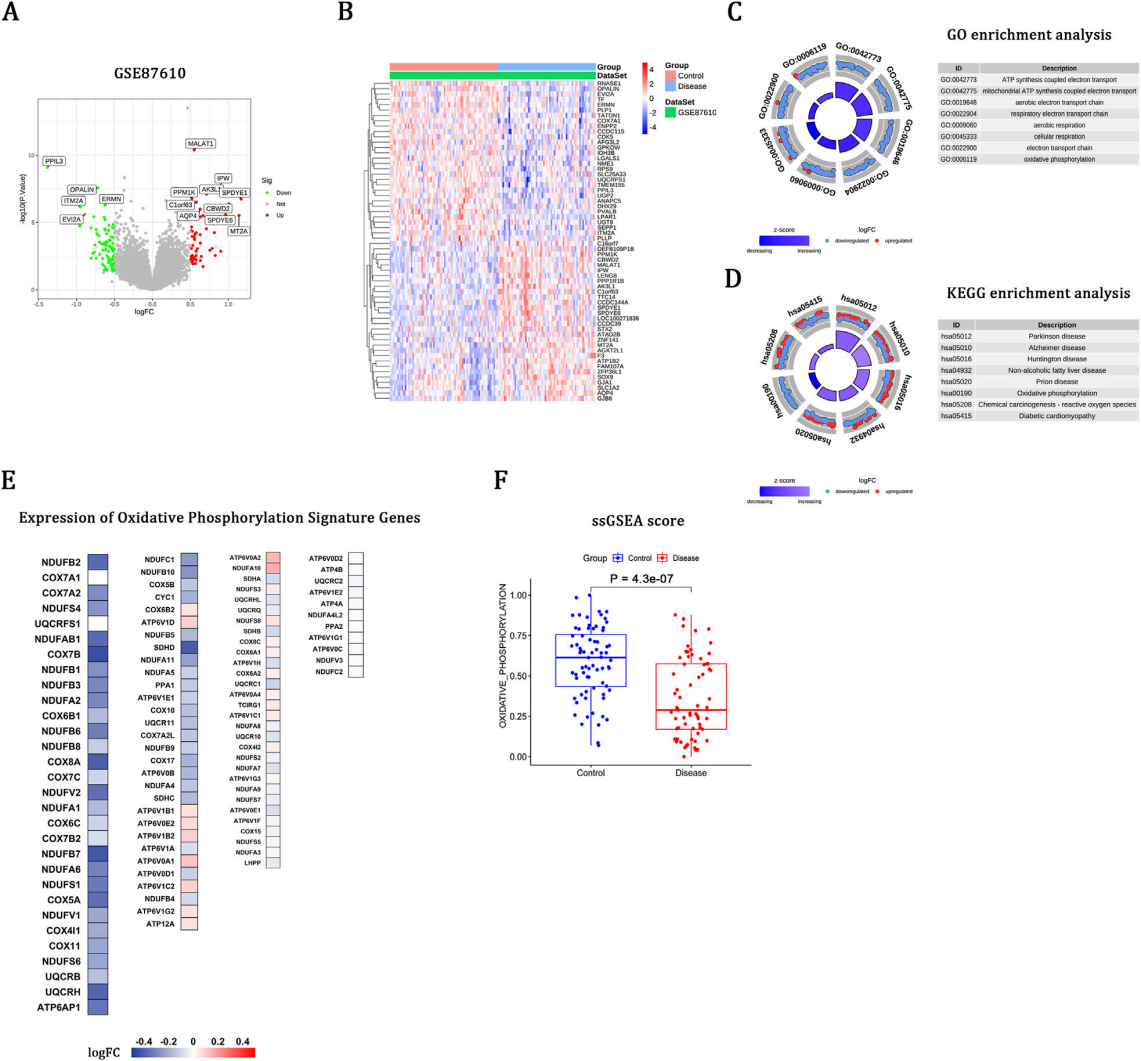
Differential expression and enrichment analyses reveal OXPHOS downregulation in schizophrenia. **(A)** Volcano plot of DEGs between schizophrenia and control brain tissues. **(B)** Heatmap of the top 30 upregulated and 30 downregulated DEGs. **(C)** GO-BP enrichment analysis. **(D)** KEGG pathway enrichment analysis. **(E)** Heatmap of OXPHOS signature genes. **(F)** Boxplot of OXPHOS enrichment scores derived from ssGSEA.

Transcriptomic profiling of the training set (GSE87610) identified 130 DEGs, including 51 upregulated and 79 downregulated genes (Figure 1A). Heatmaps illustrated the top 30 upregulated and 30 downregulated DEGs (Figure 1B). GO-BP analysis highlighted processes related to mitochondrial respiration and the electron transport chain (Figure 1C), while KEGG indicated significant enrichment in pathways associated with neurodegenerative diseases and OXPHOS (Figure 1D). A row-scaled heatmap of the OXPHOS gene set demonstrated their predominant downregulation in schizophrenia (Figure 1E). Full gene lists and detailed statistics are provided in Supplementary Table S2. This transcriptional pattern was further corroborated by ssGSEA, which demonstrated a significant reduction in OXPHOS enrichment scores in schizophrenia (Figure 1F).

### WGCNA identifies OXPHOS-associated modules linked to schizophrenia

**FIGURE 2:**
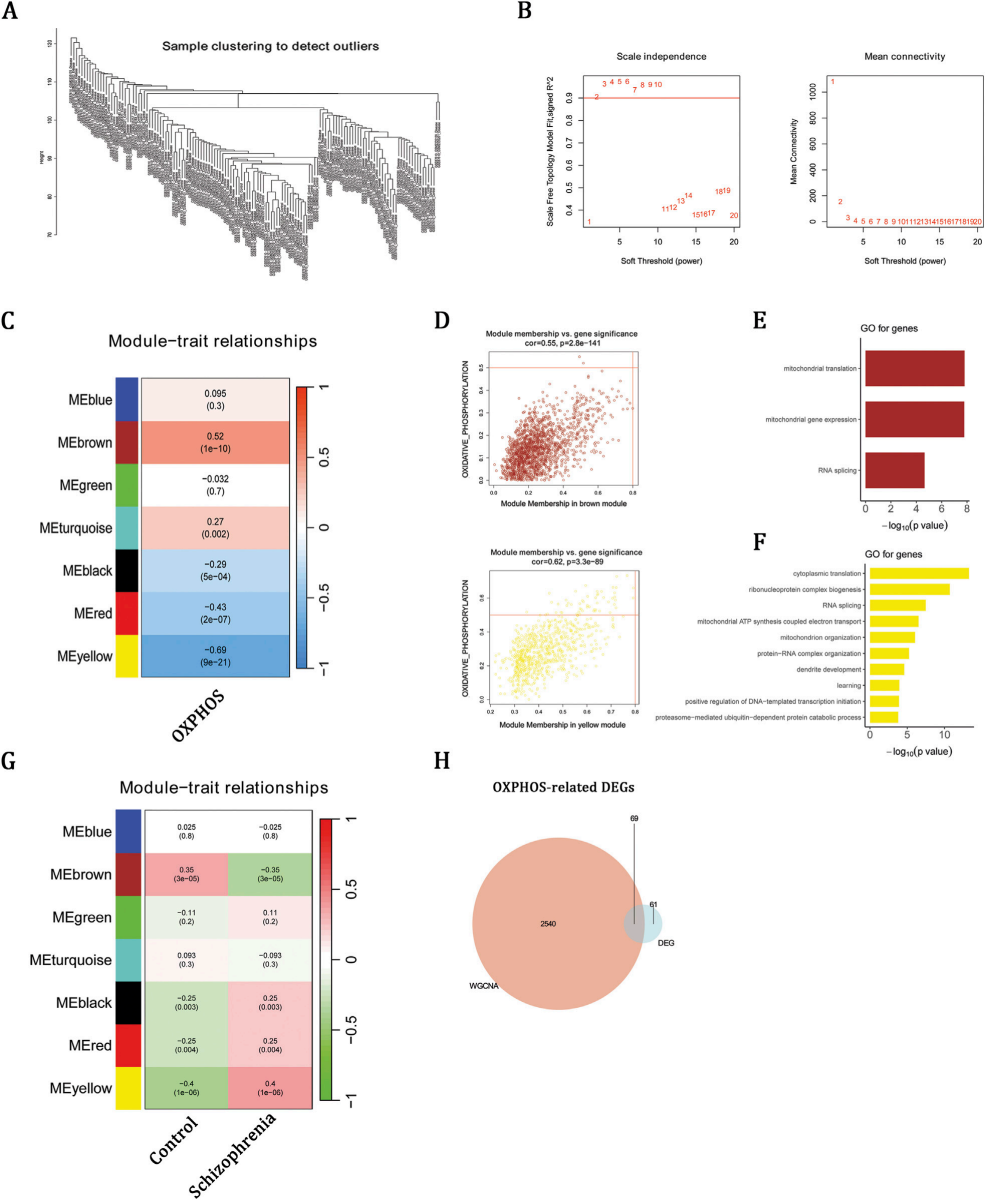
WGCNA identifies OXPHOS-associated modules linked to schizophrenia. **(A)** Sample clustering dendrogram. **(B)** Soft-thresholding power selection (β = 2). **(C,D)** Correlations between module traits and OXPHOS. **(E,F)** GO-BP enrichment of brown-module and yellow-module genes. **(G)** Correlation analysis between module traits, specifically brown and yellow, and schizophrenia status. **(H)** Venn diagram showing overlap between module genes and DEGs.

WGCNA was applied to the training set (GSE87610) to identify co-expression networks linked to OXPHOS. No outlier samples were detected (Figure 2A), and a soft-thresholding power of 2 was selected to achieve a scale-free topology (Figure 2B). The brown module showed the strongest positive correlation with OXPHOS scores, whereas the yellow module showed the strongest negative correlation (Figures 2C,D). Functional enrichment confirmed that brown-module genes (n = 1,780) were enriched in mitochondrial translation and RNA splicing, while yellow-module genes (n = 829) were enriched in respiratory-chain biogenesis and ATP synthesis (Figures 2E,F). Notably, in addition to their association with OXPHOS, the brown module was most negatively correlated with schizophrenia, whereas the yellow module was most positively correlated (Figure 2G), underscoring their close relevance to disease status. By intersecting 2,609 genes from these modules with 130 DEGs, we identified 69 OXPHOS-related DEGs (Figure 2H), which may be involved in mitochondrial dysfunction in schizophrenia.

### Machine learning prioritizes OXPHOS-associated hub genes implicated in schizophrenia

**FIGURE 3:**
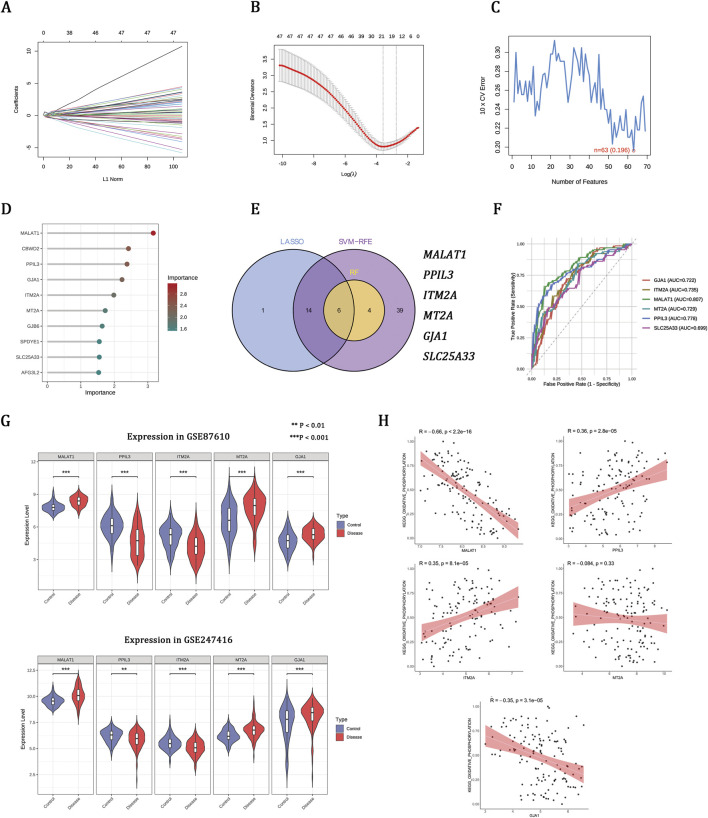
Machine learning prioritizes OXPHOS-associated hub genes implicated in schizophrenia. **(A,B)** LASSO regression showing coefficient profiles and the optimal penalty parameter (λ). **(C)** SVM-RFE curve indicating the minimum classification error. **(D)** Gene importance scores ranked by the RF model. **(E)** Venn diagram of overlapping genes identified by LASSO, SVM-RFE, and RF. **(F)** ROC curves and AUCs of the six genes. **(G)** Expression patterns of hub genes in the training set (GSE87610) and validation set (GSE247416). **(H)** Correlations between hub gene expression and OXPHOS enrichment scores in the GSE87610 dataset.

To refine disease-associated candidates, three machine learning algorithms were applied to the GSE87610 dataset, identifying 21 genes from LASSO, 63 from SVM-RFE, and 10 from RF (Figures 3A–D). Integrating these methods revealed six hub genes (Figure 3E). ROC analysis showed that five genes—MALAT1, PPIL3, ITM2A, MTA2, and GJA1—effectively distinguished schizophrenia from controls (AUC >0.70) (Figure 3F). These genes had consistent expression patterns in both the GSE87610 and GSE247416 cohorts (Figure 3G). Correlation analysis linked OXPHOS scores positively with PPIL3 and ITM2A, negatively with MALAT1 and GJA1, and not significantly with MTA2 (Figure 3H), suggesting their role in schizophrenia-related mitochondrial dysfunction.

### MK-801 schizophrenia model indicates OXPHOS impairment and gene–ATP associations

**FIGURE 4:**
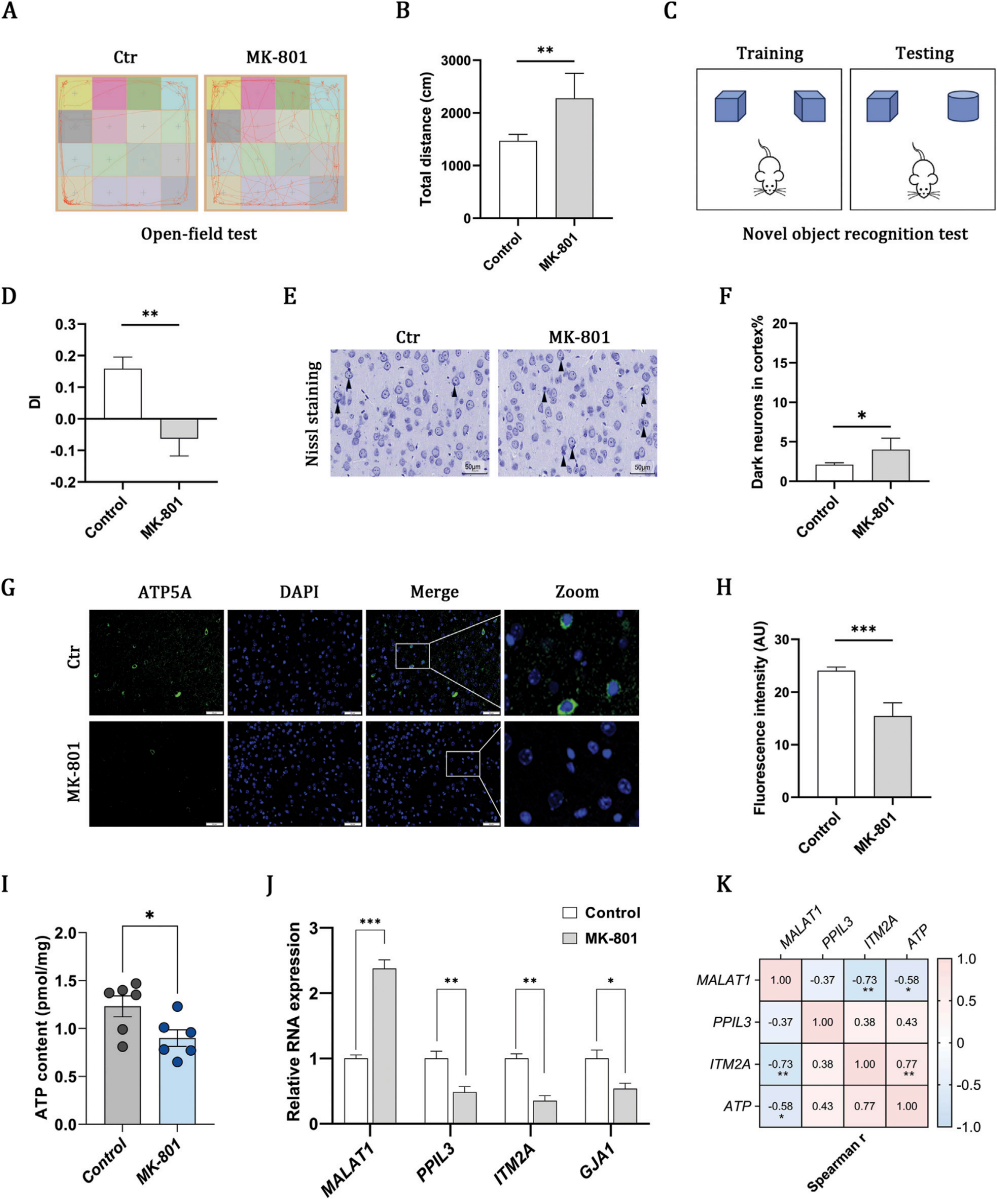
MK-801 schizophrenia model indicates OXPHOS impairment and gene–ATP associations. **(A,B)** Open-field test showing representative movement trajectories and total distance traveled. **(C,D)** Novel object recognition test and discrimination index (DI). **(E,F)** Nissl-stained cortical sections and quantification of dark neurons (scale bar, 50 μm). **(G,H)** Immunofluorescence of ATP5A (green) with DAPI-stained nuclei (blue) and quantification of fluorescence intensity (scale bar, 50 μm). **(I)** ELISA-based measurement of ATP content. **(J)** RT–qPCR of MALAT1, PPIL3, ITM2A, and GJA1 expression. **(K)** Heatmap showing associations between gene expression and ATP content. Data are presented as mean ± SEM (n = 6 per group). Statistical analyses were performed using unpaired two-tailed Student’s t-tests for two-group comparisons and Spearman correlation for association analyses. Significance: ns, P > 0.05; *P < 0.05; **P < 0.01; ***P < 0.001.

The administration of MK-801 induced marked behavioral abnormalities, including hyperlocomotion in the open field (Figures 4A,B) and impaired recognition memory in the novel object recognition test (Figures 4C,D), confirming the schizophrenia-like model. Nissl staining further identified an elevated proportion of dark neurons, which is indicative of neuronal injury (Figures 4E,F). Previous research indicates that mitochondrial OXPHOS serves as the primary source of ATP in the brain (Hall et al., 2012). Immunofluorescence staining for ATP5A revealed a reduction in fluorescence intensity (Figures 4G,H), which correlated with decreased ATP levels in the MK-801 group, as quantified by ELISA (Figure 4I). These findings collectively suggest that impaired mitochondrial oxidative phosphorylation may be involved in schizophrenia. RT-qPCR analysis corroborated the transcriptomic expression profiles of MALAT1, PPIL3, and ITM2A, as depicted in Figure 4J. Additionally, correlation analysis revealed that increased levels of MALAT1, coupled with decreased levels of PPIL3 and ITM2A, were linked to a reduction in ATP content (Figure 4K). However, it is noteworthy that the correlation with PPIL3 did not reach statistical significance.

### snRNA-seq reveals OXPHOS-enriched cell types and communication networks

**FIGURE 5:**
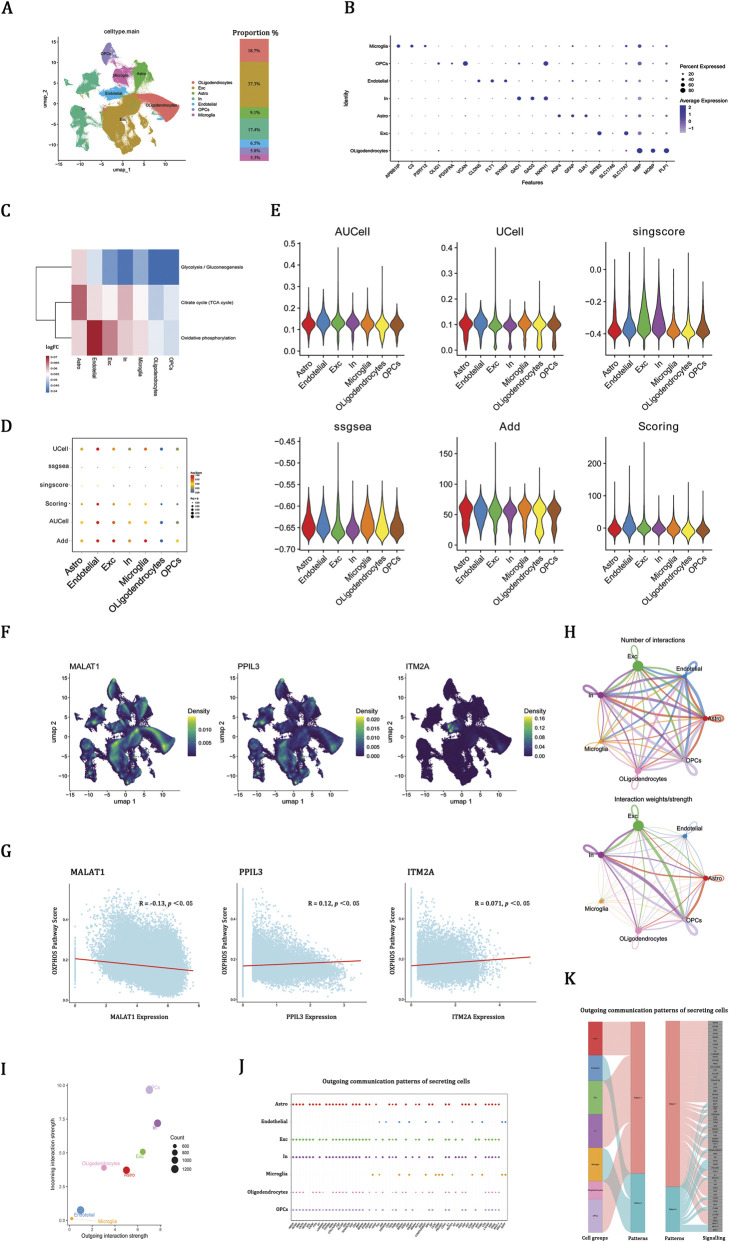
snRNA-seq reveals OXPHOS-enriched cell types and communication networks. **(A)** UMAP plot showing major cell types, including astrocytes (Astro), endothelial cells, excitatory neurons (Exc), inhibitory neurons (In), microglia, oligodendrocytes, and oligodendrocyte precursor cells (OPCs). **(B)** Canonical marker gene expression validating cell-type annotations. **(C)** Assessment of ATP-generating processes utilizing AUCell methodology. **(D,E)** OXPHOS activity across cell populations by six enrichment methods. **(F)** UMAP showing cell-type–specific expression of MALAT1, PPIL3, and ITM2A. **(G)** Correlations between hub gene expression and OXPHOS activity. **(H)** Cell–cell communication networks showing the number (left) and strength (right) of interactions among cell types. **(I)** Outgoing and incoming interaction strengths across cell types. **(J)** Bubble plot of ligand–receptor signaling contributions by cell type, with bubble size indicating contribution strength. **(K)** Sankey diagram of outgoing communication patterns.

Due to the lack of appropriate snRNA-seq datasets for schizophrenia, we analyzed the GSE247416 dataset derived from neurologically healthy adults, identifying seven key brain cell types, with excitatory neurons being the most prevalent (Figure 5A). Cell types were confirmed using marker gene expression (Figure 5B). AUCell analysis indicated that OXPHOS is the main ATP-generating pathway in the brain, surpassing glycolysis and the TCA cycle (Figure 5C). OXPHOS activity was notably higher in excitatory neurons and endothelial cells (Figures 5D,E). UMAP clustering showed MALAT1 and PPIL3 were mainly in neurons, while ITM2A was in endothelial cells (Figure 5F). Correlation analysis aligned with bulk transcriptomics, showing higher MALAT1 and lower PPIL3 and ITM2A expression linked to reduced OXPHOS scores (Figure 5G).

Having delineated the cell-type–specific OXPHOS landscape, we explored intercellular communication. Excitatory and inhibitory neurons, along with oligodendrocyte precursor cells (OPCs), showed the most frequent and strong ligand-receptor interactions (Figures 5H,I). The analysis of outgoing communication revealed that neurons function as primary signaling hubs, while endothelial cells and microglia participate in a more selective manner, predominantly through pathways associated with vascular and immune functions (Figure 5J). Sankey analysis highlighted two main signaling programs: one focused on neurotrophic, angiogenic, and extracellular matrix pathways, and the other on immune-regulatory and adhesion signals (Figure 5K). The finding that OXPHOS-enriched cell populations engage in extensive intercellular signaling suggests that mitochondrial dysfunction may extend beyond metabolism, perturbing neuronal support and neuroimmune balance.

### Pseudotemporal dynamics of OXPHOS-related hub gene

**FIGURE 6:**
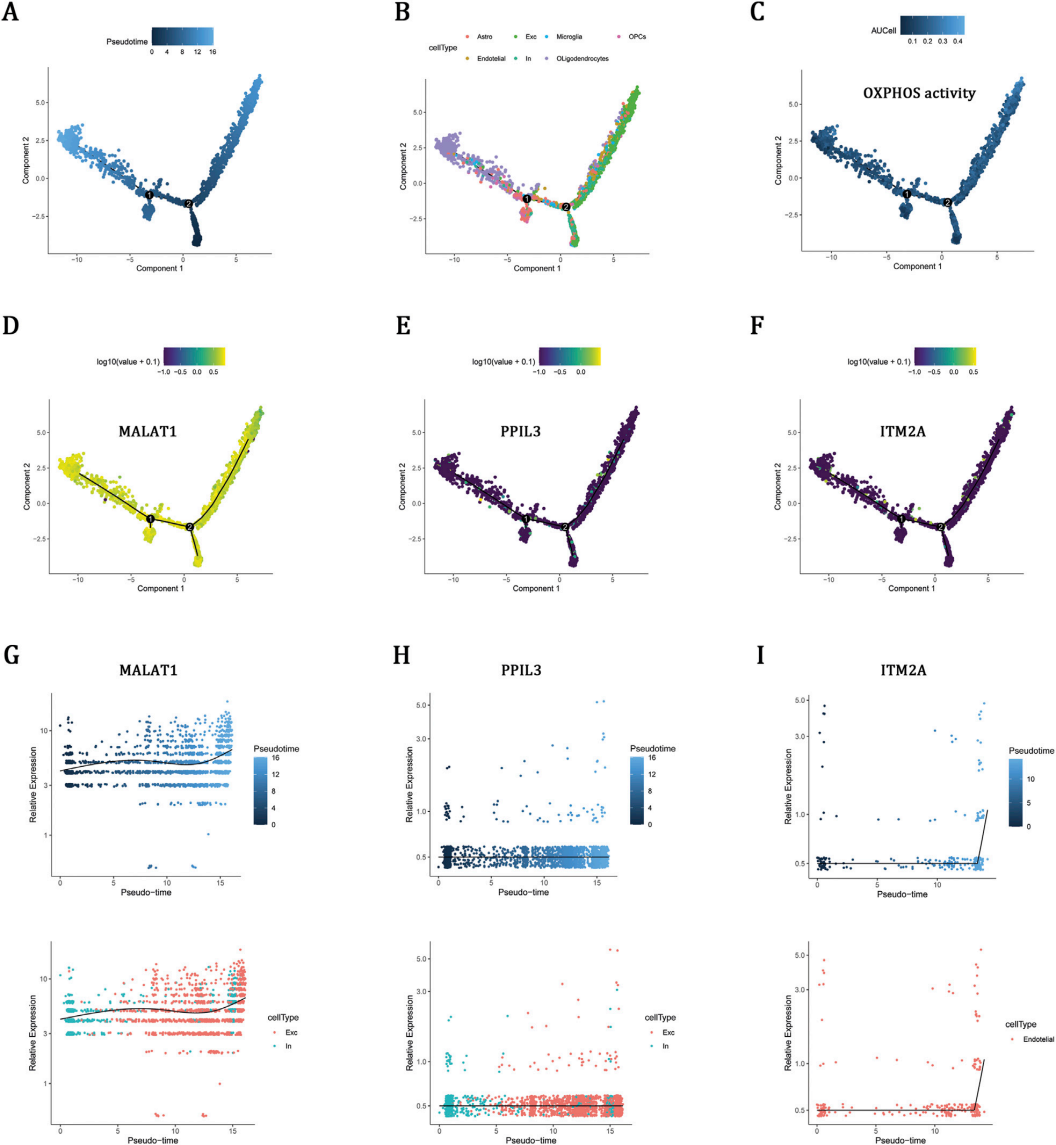
Pseudotemporal dynamics of OXPHOS-related hub gene. **(A)** Pseudotime trajectory of brain cells. **(B)** Cell-type annotation mapped onto the trajectory. **(C)** OXPHOS pathway activity (AUC scores) across pseudotime. **(D–F)** Trajectory-based expression dynamics of MALAT1, PPIL3, and ITM2A. **(G,H)** Expression patterns of MALAT1 and PPIL3 in excitatory and inhibitory neurons along pseudotime. **(I)** ITM2A expression in endothelial cells across pseudotime.

To elucidate the dynamic regulation of OXPHOS within adult brain tissue, we employed pseudotime trajectory analysis, which revealed a continuous progression with bifurcating lineages (Figure 6A). Cell-type annotation identified distinct branches for neuronal and glial populations, including astrocytes, oligodendrocytes, microglia, and OPCs, capturing biologically relevant lineage bifurcations (Figure 6B). Notably, OXPHOS activity persisted throughout pseudotime, suggesting sustained metabolic involvement in cell state transitions (Figure 6C). Expression analysis of OXPHOS-related key genes further showed that MALAT1 maintained high expression levels at both early and late pseudotime stages, suggesting a sustained functional role. In contrast, PPIL3 and ITM2A exhibited relatively lower expression levels, implying potential auxiliary functions (Figures 6D–F). Given their distinct cell-type-specific expression patterns, MALAT1 and PPIL3 enriched in neurons, and ITM2A predominantly in endothelial cells (Figure 5F), we subsequently conducted cell type–specific pseudotime analyses. MALAT1 and PPIL3 were preferentially expressed in inhibitory neurons during early pseudotime and in excitatory neurons during later stages, indicating stage- and cell-type–specific regulation of neuronal states (Figures 6G,H). Conversely, ITM2A expression progressively increased in endothelial cells over pseudotime, pointing to its involvement in endothelial cell state transitions (Figure 6I).

## Discussion

Mitochondrial OXPHOS dysfunction has been increasingly linked to schizophrenia, but its molecular basis remains poorly defined. Using an integrative approach combining bulk transcriptomics, snRNA-seq, and an MK-801 mouse model, we observed reduced OXPHOS in schizophrenia and identified three candidate genes—MALAT1, PPIL3, and ITM2A—associated with this process. The findings indicate a possible link between OXPHOS dysfunction and schizophrenia, thus offering a basis for further mechanistic investigations and the exploration of targeted therapeutic interventions.

Clinical investigations have documented diminished activities of respiratory chain complexes I and IV in blood samples from patients (Morén et al., 2025). Corroborating these findings, Fizíková et al. demonstrated that disruptions in the tricarboxylic acid cycle and oxidative phosphorylation (OXPHOS) within neurons and astrocytes adversely affect energy metabolism and neurotransmission (Fizíková et al., 2023). At the molecular level, transcriptomic analyses of cortical regions have revealed a downregulation of OXPHOS-related genes, particularly within networks associated with visuospatial working memory, thereby further associating mitochondrial dysfunction with cognitive impairment in schizophrenia (Kimoto et al., 2022). In addition to compromised bioenergetics, dysfunction in OXPHOS has been associated with secondary disturbances, such as oxidative stress, calcium dysregulation, and aberrant inflammatory signaling. These disturbances are widely recognized as pathological hallmarks of schizophrenia (Ermakov et al., 2021; De Simone et al., 2023). Both genetic and developmental evidence further underscore the pivotal role of OXPHOS metabolism in schizophrenia. Genome-wide association studies have demonstrated a significant enrichment of nuclear-encoded mitochondrial genes, while the 22q11.2 deletion syndrome, one of the most penetrant genetic risk factors, induces a metabolic shift from OXPHOS to glycolysis (Napoli et al., 2015). Therapeutic evidence increasingly implicates mitochondrial pathways. Protein profiling conducted on murine models treated with chlorpromazine, clozapine, or quetiapine has demonstrated a significant alteration in mitochondrial proteins, particularly within the OXPHOS subunits. This suggests that modulation of mitochondrial metabolism may play a role in the therapeutic efficacy of these agents (Ji et al., 2009).

In agreement with prior studies, our bulk transcriptomic analysis revealed significantly reduced OXPHOS enrichment scores in schizophrenia. Consistently, MK-801–treated mice exhibited diminished ATP5A fluorescence and ATP concentrations, supporting impaired mitochondrial function. Complementary snRNA-seq analysis further corroborated OXPHOS as the principal ATP-generating pathway in the brain, in line with previous evidence (Hall et al., 2012). However, it should be noted that MK-801 has also been reported to affect glycolysis, particularly in oligodendrocytes (Guest et al., 2015), raising the possibility that the observed ATP reductions reflect broader metabolic disturbances. Taken together, while glycolytic alterations cannot be excluded, the convergence of bulk transcriptomic, snRNA-seq, and *in vivo* data highlights OXPHOS dysfunction as a reproducible feature that may contribute to schizophrenia pathophysiology. To probe its molecular basis in schizophrenia, we applied complementary methodologies with multi-level validation, which converged on three genes—MALAT1, PPIL3, and ITM2A—that consistently correlated with OXPHOS activity.

Metastasis-associated lung adenocarcinoma transcript 1 (MALAT1) is a ubiquitously expressed nuclear long non-coding RNA (lncRNA) (Gutschner et al., 2013). Elevated MALAT1 levels have been detected in the peripheral blood of patients with schizophrenia and correlate positively with disease duration (Fallah et al., 2019), yet its role in the nervous system remains unclear. In cardiomyocytes exposed to high glucose *in vitro* and in myocardial tissue from mice with diabetic cardiomyopathy *in vivo*, MALAT1 expression is upregulated. Knockdown of MALAT1 in these settings alleviates oxidative stress and mitochondrial damage, suggesting a detrimental effect on OXPHOS (Wang et al., 2023). In immune cells, MALAT1 expression is inversely associated with genes involved in cell division, OXPHOS, and cytokine responses during T cell activation (Dey et al., 2023). Silencing MALAT1 enhances mitochondrial OXPHOS in IL-4–treated macrophages (Cui et al., 2019). By contrast, in prostate cancer cells, MALAT1 silencing downregulates ME3, PDK1, PDK3, and choline kinase—enzymes critical for OXPHOS (Nanni et al., 2020). In hepatocellular carcinoma, MALAT1 localizes to mitochondria and interacts with multiple mitochondrial DNA (mtDNA) loci (D-loop, COX2, ND3, CYTB). MALAT1 knockdown could alter mtDNA methylation and transcription, leading to structural disruption, impaired OXPHOS, and reduced ATP production (Zhao et al., 2021). Collectively, these findings suggest that MALAT1 may have either promotive or inhibitory effects on OXPHOS, depending on the cell type and context. In schizophrenia, we found that MALAT1 is highly expressed across multiple cell types, including neurons, and negatively correlates with OXPHOS. This association suggests that elevated MALAT1 may impair mitochondrial energy production in neurons, thereby contributing to synaptic dysfunction and cognitive deficits.

Peptidyl-prolyl isomerase-like 3 (PPIL3), also known as cyclophilin J (CyPJ), is a spliceosome-associated cyclophilin involved in mRNA splicing and a member of the cyclophilin (CyP) family. CyPs, particularly CyPD, have been shown to facilitate the assembly of ATP synthase into higher-order supercomplexes, thereby stabilizing the respiratory chain and enhancing OXPHOS efficiency (Coluccino et al., 2023). Overexpression of CyPB mitigates oxidative stress by upregulating antioxidant enzymes such as manganese superoxide dismutase and catalase, thereby protecting against MPP^+^-induced mitochondrial dysfunction and neurotoxicity (Oh et al., 2016); in human kidney-2 cells, it also reduces reactive oxygen species and restores mitochondrial function under aldosterone stress (Wang et al., 2016). In our study, we observed a significant enrichment of PPIL3 in excitatory neurons, accompanied by a positive correlation with OXPHOS. Given the mitochondrial roles of other cyclophilins, we hypothesize that reduced PPIL3 expression may impair neuronal energy metabolism and disrupt the excitation–inhibition balance.

Integral membrane protein 2A (ITM2A) is a transmembrane protein predominantly expressed in brain endothelial cells and widely recognized as a marker of the blood–brain barrier (BBB) (Zhang et al., 2016; Cegarra et al., 2022). BBB abnormalities have been documented in individuals with schizophrenia (Najjar et al., 2017). Experimental evidence further indicates that mitochondrial respiration supports BBB integrity, whereas impaired OXPHOS in brain endothelial cells compromises barrier function and promotes neuroinflammation (Lee et al., 2022). In our study, ITM2A was selectively expressed in brain endothelial cells and positively correlated with OXPHOS, although its precise role in the central nervous system remains undefined. Autophagy is integral to the maintenance of OXPHOS, as it facilitates the removal of damaged mitochondria and recycles substrates for the tricarboxylic acid cycle (García-Miranda et al., 2024). Indeed, ITM2A has been shown to regulate autophagy in a context-dependent manner: in breast cancer cells, it enhances mTOR-dependent autophagy to inhibit tumor growth (Zhou et al., 2019), whereas in HEK293 cells, its overexpression disrupts vacuolar ATP synthase and blocks autophagic flux (Namkoong et al., 2015). Given the specific enrichment of ITM2A in brain endothelial cells, we propose that its dysregulation may perturb the autophagy–OXPHOS axis, thereby compromising BBB integrity and ultimately aggravating neuronal and synaptic dysfunction.

This study has several limitations. First, although we identified a link between OXPHOS dysfunction and schizophrenia, causality remains unresolved; genetic or pharmacological interventions will be needed to determine whether mitochondrial abnormalities are drivers or consequences of the disease. Second, the absence of schizophrenia-specific snRNA-seq data limited validation of cell type–specific differential expression; future patient-derived datasets will enable more precise characterization. Third, the MK-801 model captures only a subset of schizophrenia features and cannot reflect the full complexity of the disorder; validation in genetic or iPSC-based models will improve translational relevance. Finally, the associations of MALAT1, PPIL3, and ITM2A with OXPHOS are correlative, and mechanistic studies in neuronal and endothelial contexts are required to establish causality.

## Conclusion

In conclusion, through the integration of bulk transcriptomics, snRNA-seq, and *in vivo* validation, we propose a potential link between OXPHOS dysfunction and schizophrenia. The genes MALAT1, PPIL3, and ITM2A emerge as candidate regulators in this process, meriting further investigation into their causal roles and therapeutic significance.

## Data Availability

The original contributions presented in the study are included in the article/Supplementary Material, further inquiries can be directed to the corresponding authors. Publicly available datasets were analyzed in this study. These data can be found here: https://www.ncbi.nlm.nih.gov/geo/query/acc.cgi?acc=GSE87610, https://www.ncbi.nlm.nih.gov/geo/query/acc.cgi?acc=GSE53987, https://www.ncbi.nlm.nih.gov/geo/query/acc.cgi?acc=GSE247416.
