# Supplementary material for: Multi-omics identification and validation of oxidative phosphorylation–related hub genes in schizophrenia
Source: Front Genet. 2025 Oct 23;16:1690947. doi: 10.3389/fgene.2025.1690947 (PMC12588580; doi:10.3389/fgene.2025.1690947)
Supplement: Supplementary file 1 [file Table1.docx]

**Supplementary TABLE 1.** List of 132 OXPHOS marker genes

| ATP12A | ATP6V0A1 | ATP6V1G3 | COX7B2 | NDUFA10 | NDUFB8 | SDHD |
| --- | --- | --- | --- | --- | --- | --- |
| ATP4A | ATP6V0A2 | ATP6V1H | COX7C | NDUFA11 | NDUFB9 | TCIRG1 |
| ATP4B | ATP6V0A4 | COX10 | COX8A | NDUFA2 | NDUFC1 | UQCR10 |
| ATP5F1A | ATP6V0B | COX11 | COX8C | NDUFA3 | NDUFC2 | UQCR10P1 |
| ATP5F1B | ATP6V0C | COX15 | CYC1 | NDUFA4 | NDUFS1 | UQCR11 |
| ATP5F1C | ATP6V0D1 | COX17 | LHPP | NDUFA4L2 | NDUFS2 | UQCRB |
| ATP5F1D | ATP6V0D2 | COX4I1 | MT-ATP6 | NDUFA5 | NDUFS3 | UQCRC1 |
| ATP5F1E | ATP6V0E1 | COX4I2 | MT-ATP8 | NDUFA6 | NDUFS4 | UQCRC2 |
| ATP5MC1 | ATP6V0E2 | COX5A | MT-CO1 | NDUFA7 | NDUFS5 | UQCRFS1 |
| ATP5MC1P5 | ATP6V1A | COX5B | MT-CO2 | NDUFA8 | NDUFS6 | UQCRH |
| ATP5MC2 | ATP6V1B1 | COX6A1 | MT-CO3 | NDUFA9 | NDUFS7 | UQCRHL |
| ATP5MC3 | ATP6V1B2 | COX6A2 | MT-CYB | NDUFAB1 | NDUFS8 | UQCRQ |
| ATP5ME | ATP6V1C1 | COX6B1 | MT-ND1 | NDUFB1 | NDUFV1 |  |
| ATP5MF | ATP6V1C2 | COX6B2 | MT-ND2 | NDUFB10 | NDUFV2 |  |
| ATP5MG | ATP6V1D | COX6C | MT-ND3 | NDUFB2 | NDUFV3 |  |
| ATP5PB | ATP6V1E1 | COX6CP3 | MT-ND4 | NDUFB3 | PPA1 |  |
| ATP5PD | ATP6V1E2 | COX7A1 | MT-ND4L | NDUFB4 | PPA2 |  |
| ATP5PF | ATP6V1F | COX7A2 | MT-ND5 | NDUFB5 | SDHA |  |
| ATP5PO | ATP6V1G1 | COX7A2L | MT-ND6 | NDUFB6 | SDHB |  |
| ATP6AP1 | ATP6V1G2 | COX7B | NDUFA1 | NDUFB7 | SDHC |  |
