# Supplementary material for: Multi-omics identification and validation of oxidative phosphorylation–related hub genes in schizophrenia
Source: Front Genet. 2025 Oct 23;16:1690947. doi: 10.3389/fgene.2025.1690947 (PMC12588580; doi:10.3389/fgene.2025.1690947)
Supplement: Supplementary file 2 [file Table2.docx]

**Supplementary TABLE 2.** Expression profiles of OXPHOS-related genes in schizophrenia

| **id** | **logFC** | **AveExpr** | **t** | **P.Value** | **B** |
| --- | --- | --- | --- | --- | --- |
| NDUFB2 | -0.37244 | 10.9617 | -5.8143 | 4.08E-08 | 8.225403 |
| COX7A1 | -0.77009 | 9.104297 | -4.8206 | 3.75E-06 | 4.119284 |
| COX7A2 | -0.2845 | 8.228031 | -4.76772 | 4.7E-06 | 3.915052 |
| NDUFS4 | -0.27017 | 5.563133 | -4.70801 | 6.05E-06 | 3.686339 |
| UQCRFS1 | -0.51463 | 8.387909 | -4.50634 | 1.4E-05 | 2.929086 |
| NDUFAB1 | -0.37373 | 7.62537 | -4.34731 | 2.67E-05 | 2.348953 |
| COX7B | -0.44178 | 8.803087 | -4.23732 | 4.13E-05 | 1.956771 |
| NDUFB1 | -0.27907 | 9.972312 | -3.97507 | 0.000113 | 1.052631 |
| NDUFB3 | -0.29633 | 7.374624 | -3.81641 | 0.000204 | 0.527511 |
| NDUFA2 | -0.29774 | 7.106198 | -3.70655 | 0.000304 | 0.173912 |
| COX6B1 | -0.17161 | 10.81684 | -3.63041 | 0.000399 | -0.06629 |
| NDUFB6 | -0.32943 | 7.892264 | -3.36353 | 0.000998 | -0.87572 |
| NDUFB8 | -0.12573 | 10.33126 | -3.27016 | 0.001359 | -1.14671 |
| COX8A | -0.40245 | 7.971047 | -3.26513 | 0.001382 | -1.16114 |
| COX7C | -0.10247 | 11.38387 | -3.25977 | 0.001406 | -1.17648 |
| NDUFV2 | -0.36156 | 7.291446 | -3.25048 | 0.00145 | -1.20302 |
| NDUFA1 | -0.17021 | 10.5163 | -3.21022 | 0.001652 | -1.31728 |
| COX6C | -0.11105 | 8.597813 | -3.18078 | 0.001817 | -1.40007 |
| COX7B2 | -0.08863 | 2.016224 | -3.17562 | 0.001847 | -1.4145 |
| NDUFB7 | -0.4239 | 7.219173 | -3.15904 | 0.001948 | -1.46079 |
| NDUFA6 | -0.3167 | 8.114856 | -3.12848 | 0.002147 | -1.54552 |
| NDUFS1 | -0.32984 | 7.285483 | -3.01745 | 0.00304 | -1.84747 |
| COX5A | -0.3636 | 7.755474 | -2.99418 | 0.003266 | -1.90954 |
| NDUFV1 | -0.21396 | 8.69421 | -2.9451 | 0.003794 | -2.03909 |
| COX4I1 | -0.21066 | 8.388083 | -2.77394 | 0.006311 | -2.47619 |
| COX11 | -0.22095 | 5.357306 | -2.70337 | 0.007733 | -2.64968 |
| NDUFS6 | -0.22479 | 8.904881 | -2.68994 | 0.008035 | -2.68223 |
| UQCRB | -0.15471 | 8.028971 | -2.66071 | 0.008728 | -2.7526 |
| UQCRH | -0.38794 | 8.16478 | -2.65789 | 0.008798 | -2.75936 |
| ATP6AP1 | -0.34272 | 6.095038 | -2.63477 | 0.009389 | -2.81447 |
| NDUFC1 | -0.25584 | 7.78496 | -2.62733 | 0.009586 | -2.8321 |
| NDUFB10 | -0.26875 | 8.081196 | -2.62213 | 0.009726 | -2.84441 |
| COX5B | -0.14644 | 9.78377 | -2.47739 | 0.01445 | -3.17808 |
| CYC1 | -0.19957 | 7.424928 | -2.47168 | 0.014673 | -3.1909 |
| COX6B2 | 0.053226 | 2.743609 | 2.438991 | 0.016007 | -3.26372 |
| ATP6V1D | 0.090262 | 9.647689 | 2.354276 | 0.019978 | -3.44833 |
| NDUFB5 | -0.17753 | 9.299248 | -2.34608 | 0.020405 | -3.46586 |
| SDHD | -0.41305 | 5.405107 | -2.30048 | 0.022931 | -3.56244 |
| NDUFA11 | -0.23792 | 6.923536 | -2.2621 | 0.025264 | -3.64236 |
| NDUFA5 | -0.11811 | 10.08016 | -2.11839 | 0.035946 | -3.93058 |
| PPA1 | -0.13108 | 9.969857 | -2.11543 | 0.036202 | -3.93632 |
| ATP6V1E1 | -0.11717 | 9.705354 | -2.08411 | 0.039006 | -3.99673 |
| COX10 | -0.16139 | 2.885783 | -2.0601 | 0.04128 | -4.04245 |
| UQCR11 | -0.1615 | 6.111672 | -2.04305 | 0.042963 | -4.07462 |
| COX7A2L | -0.14412 | 8.366403 | -1.94598 | 0.053704 | -4.25301 |
| NDUFB9 | -0.11811 | 7.328852 | -1.80785 | 0.072822 | -4.49266 |
| COX17 | -0.19123 | 8.387667 | -1.75198 | 0.082014 | -4.58485 |
| ATP6V0B | -0.17986 | 6.420179 | -1.71626 | 0.088374 | -4.64234 |
| NDUFA4 | -0.14368 | 8.649302 | -1.68951 | 0.093396 | -4.68465 |
| SDHC | -0.17142 | 6.940075 | -1.66803 | 0.097592 | -4.71815 |
| ATP6V1B1 | 0.056761 | 2.095545 | 1.554408 | 0.122393 | -4.88858 |
| ATP6V0E2 | 0.071727 | 4.862734 | 1.499968 | 0.135922 | -4.96614 |
| ATP6V1B2 | 0.092639 | 9.849621 | 1.479052 | 0.141421 | -4.99523 |
| ATP6V1A | -0.08186 | 9.364508 | -1.46367 | 0.145573 | -5.01636 |
| ATP6V0A1 | 0.109949 | 7.662092 | 1.391321 | 0.166382 | -5.11292 |
| ATP6V0D1 | -0.11636 | 7.681713 | -1.31939 | 0.189239 | -5.20421 |
| ATP6V1C2 | 0.088006 | 2.404654 | 1.274129 | 0.204773 | -5.25923 |
| NDUFB4 | -0.09439 | 6.264173 | -1.21731 | 0.225577 | -5.32564 |
| ATP6V1G2 | 0.060167 | 9.905439 | 1.214638 | 0.226592 | -5.3287 |
| ATP12A | 0.058423 | 3.559586 | 1.198195 | 0.232908 | -5.34732 |
| ATP6V0A2 | 0.140633 | 4.264158 | 1.181335 | 0.239515 | -5.36617 |
| NDUFA10 | 0.157359 | 7.230389 | 1.159018 | 0.248464 | -5.39071 |
| SDHA | -0.09738 | 6.652113 | -1.15535 | 0.249958 | -5.3947 |
| NDUFS3 | 0.04794 | 6.429125 | 1.139202 | 0.256606 | -5.41211 |
| UQCRHL | -0.07326 | 7.072939 | -1.13775 | 0.257211 | -5.41367 |
| UQCRQ | -0.05312 | 5.116915 | -1.08598 | 0.279394 | -5.46781 |
| NDUFS8 | 0.058751 | 5.571075 | 1.042329 | 0.299094 | -5.51154 |
| SDHB | -0.10006 | 7.313413 | -1.03814 | 0.301032 | -5.51564 |
| COX8C | 0.030552 | 2.434665 | 1.000937 | 0.318621 | -5.55138 |
| COX6A1 | 0.03101 | 7.437385 | 0.975502 | 0.33103 | -5.57507 |
| ATP6V1H | -0.0865 | 7.109947 | -0.96393 | 0.336779 | -5.58565 |
| COX6A2 | 0.035283 | 2.995229 | 0.943531 | 0.347069 | -5.60399 |
| UQCRC1 | -0.10876 | 5.206132 | -0.93776 | 0.350019 | -5.60912 |
| ATP6V0A4 | 0.033913 | 2.842762 | 0.841488 | 0.40154 | -5.68995 |
| TCIRG1 | 0.046857 | 3.790143 | 0.767653 | 0.444014 | -5.74608 |
| ATP6V1C1 | 0.039097 | 7.654079 | 0.701405 | 0.48424 | -5.7921 |
| NDUFA8 | -0.03324 | 5.991229 | -0.67886 | 0.498371 | -5.80682 |
| UQCR10 | -0.07358 | 7.976471 | -0.67113 | 0.503265 | -5.81176 |
| COX4I2 | 0.029508 | 2.693734 | 0.655472 | 0.513263 | -5.82159 |
| NDUFS2 | -0.03752 | 5.163657 | -0.62172 | 0.535156 | -5.84199 |
| NDUFA7 | -0.07774 | 5.383274 | -0.59582 | 0.552277 | -5.85692 |
| ATP6V1G3 | 0.019481 | 2.232629 | 0.591845 | 0.554929 | -5.85916 |
| NDUFA9 | -0.03406 | 10.093 | -0.56942 | 0.570003 | -5.87149 |
| NDUFS7 | -0.04165 | 3.802639 | -0.5338 | 0.594344 | -5.89011 |
| ATP6V0E1 | -0.08174 | 5.494022 | -0.42915 | 0.668486 | -5.93787 |
| ATP6V1F | -0.02676 | 9.449675 | -0.40284 | 0.687694 | -5.94825 |
| COX15 | -0.0345 | 4.206025 | -0.39785 | 0.69136 | -5.95014 |
| NDUFS5 | -0.01737 | 6.583733 | -0.38935 | 0.697621 | -5.95331 |
| NDUFA3 | 0.018509 | 9.372698 | 0.380856 | 0.703899 | -5.95642 |
| LHPP | -0.05195 | 3.926136 | -0.36096 | 0.718683 | -5.96342 |
| ATP6V0D2 | -0.00747 | 2.194954 | -0.35524 | 0.722958 | -5.96536 |
| ATP4B | -0.00898 | 2.44298 | -0.2908 | 0.771642 | -5.98511 |
| UQCRC2 | -0.0234 | 9.030241 | -0.2848 | 0.776227 | -5.98675 |
| ATP6V1E2 | -0.02348 | 4.043622 | -0.21114 | 0.83309 | -6.00409 |
| ATP4A | 0.009166 | 3.325913 | 0.207435 | 0.835978 | -6.00482 |
| NDUFA4L2 | 0.006094 | 2.330653 | 0.155789 | 0.876428 | -6.01373 |
| PPA2 | 0.007878 | 4.031428 | 0.136086 | 0.891953 | -6.01646 |
| ATP6V1G1 | -0.00984 | 5.062904 | -0.12554 | 0.900278 | -6.01777 |
| ATP6V0C | 0.003591 | 9.868756 | 0.058317 | 0.953581 | -6.02364 |
| NDUFV3 | 0.004631 | 5.692315 | 0.047177 | 0.962441 | -6.02419 |
| NDUFC2 | 0.001169 | 7.740403 | 0.014119 | 0.988756 | -6.02516 |
